# Citric Acid-Treated Zeolite Y (CY)/Zeolite Beta Composites as Supports for Vacuum Gas Oil Hydrocracking Catalysts: High Yield Production of Highly-Aromatic Heavy Naphtha and Low-BMCI Value Tail Oil

**DOI:** 10.3389/fchem.2019.00705

**Published:** 2019-10-29

**Authors:** Qiang Wei, Jiarui Zhang, Xiaodong Liu, Pengfei Zhang, Shuqin Wang, Yan Wang, Zhenli Zhang, Tao Zhang, Yasong Zhou

**Affiliations:** ^1^State Key Laboratory of Heavy Oil Processing, China University of Petroleum, Beijing, China; ^2^Petrochemical Research Institute, China National Petroleum Corporation, Beijing, China

**Keywords:** zeolite Y, zeolite beta, hydrocracking, catalyst, composite zeolite

## Abstract

Citric acid-treated zeolite Y (CY) and zeolite beta were mechanically mixed to obtain composite zeolites (CY-Beta) with various zeolite beta contents. The composite zeolites were used as the acid components of hydrocracking catalyst supports. The physical and chemical properties of the supports and catalysts were analyzed by N_2_ adsorption-desorption, XRD, SEM, and NH_3_-TPD. The mechanical mixing of CY and zeolite beta does not destroy the textual properties of the original zeolites. However, the acidity of the composite zeolite does not fit the linearly calculated value of the two zeolites because some of the acid sites are covered or reacted with other acid sites during the mixing process. In addition, weak acid sites favor the high yield of tail oil with low BMCI value. Compared with the CY-based and beta-based catalysts, the conversion and light oil yield of the CY-Beta-based catalyst was increased. The conversion, light oil yield, and petrochemical yield of the Ni-W/20CY-Beta(20)/ASA catalyst are 78.15, 65.0, and 83.7%, respectively. The BMCI value of the tail oil is 4.7, and the aromatic potential content (APC) of heavy naphtha (boiling point 65–177°C) is 42%. The 1,500 h pilot plant test of Ni-W/20CY-Beta(20)/ASA at 350°C, 7.0 MPa, 2.0 h^−1^ LHSV, and 800 H_2_/oil (v/v) shows that the activity remains stable during the 1,500 h evaluation. The heavy naphtha (APC about 41.0) yield of 41.2 illustrates that the catalyst has the ability to aromatize and cyclize the light fractions. The yield of diesel is about 25% with a cetane index (CI) of 59.2; the frozen point is lower than −45°C, and the cold filter plugging point is −35°C, demonstrating the isomerization performance for middle distillations. The yield of tail oil is 14.9% with a BMCI of 4.4, showing the high hydrogenation performance of the catalyst to transform the un-cracked tail oil to saturated hydrocarbon in order to reduce the BMCI value.

## Introduction

With the depleting conventional crude supply and increasing heavy crude production, large amounts of low-quality heavy petroleum fractions require more severe upgrading and processing to produce clean transportation fuels (Breysse et al., 2003; Song, 2003). In refinery operation, catalytic hydrocracking is the process of choice for converting heavy petroleum fractions into clean transportation fuels and feedstocks for the petrochemical industry (Minderhoud et al., 1999). Among the hydrocracking products, heavy naphtha (65°-177°C) and tail oil (320°C^+^ distillation) are high-value petrochemical feedstocks. Heavy naphtha with a high aromatic potential content (APC) (Han et al., 2014) is commonly used as catalytic reforming feedstock to produce aromatics. Tail oil with a low Bureau of Mines Correlation Index (BMCI) value (Nyathi et al., 2012) is used as steam-cracking feedstock to produce ethane.

The key reactions in the hydrocracking of heavy feedstocks include: (a) partial hydrogenation of polycyclic aromatics followed by ring openings to generate substituted aromatics; (b) hydrogenation of substituted aromatics to naphthenic structures; and (c) naphthenic ring openings and lateral chain elimination to generate linear and branched paraffins and olefins with the boiling point ranges of naphtha and medium distillates (Benazzi et al., 2003; Ancheyta et al., 2005). The commonly used commercial hydrocracking catalysts are alumina and zeolite-supported cobalt (Co)/nickel (Ni)-promoted molybdenum (Mo) or/and tungsten (W) sulfide bi-functional catalysts (Sato et al., 2001; Morawski and Mosio-Mosiewski, 2006; Tayeb et al., 2010). The hydrogenation reactions occur at the active metal sites, and the C–C bond cleavages occur at the acid sites of the catalyst (Qiu et al., 2008; Francis et al., 2011). The selectivity for the light fraction (naphtha) over the hydrocracked product is known to be dependent on the catalytic cracking activity and hydrogenation activity (Ding et al., 2007; Francis et al., 2011). The catalytic cracking activity is affected by the acidity of the acidic components of the catalyst support, which comprises various types of zeolites (Sato et al., 2001; Ohshio et al., 2004). Zeolite Y is a commonly used acidic catalyst support due to its pore structure, acidity, and thermal and hydrothermal stability in refinery applications (Kuznetsov, 2003; Alsobaai et al., 2007b). Zeolite beta is another commonly used acidic catalyst support that has a high SiO_2_/Al_2_O_3_ ratio and exhibits a three-dimensional (3-D) porous structure. Chemical treatment can be used to achieve desirable properties such as high hydroisomerization, low hydrogen-transfer capacity, and low catalyst deactivation in zeolite beta in order to carry out the desirable catalytic reactions (Liu et al., 2009; Alzaid and Smith, 2013).

Since hydrocracking reactions occur at the acid sites of zeolite, the strongly acid sites, and small pores of zeolite have dominant impacts on the reaction product distribution, especially the light oil yield (Laxmi Narasimhan et al., 2004; Ferraz et al., 2010; Francis et al., 2011). Therefore, the objective of applying hydrocracking technology to produce petrochemical feedstocks is to maximize the productions of heavy naphtha with a high APC and tail oil with a low BMCI (Zhao et al., 2011). To achieve the process objectives, hydrocracking reactions must be controlled to occur at different acid sites (González et al., 2011, 2012). To obtain high yields of high-APC heavy naphtha, a high isomerization catalytic activity is needed. To obtain low-BMCI tail oils, a high hydrogenation catalytic activity is needed to produce saturated linear hydrocarbons followed by C–C bond cleavages at the cracking active sites without isomerization reactions (Qiu et al., 2008; Ferraz et al., 2010). Therefore, the use of a single zeolite as a catalyst support cannot provide sufficient acid sites to facilitate two different reactions. As a result, the typical hydrocracking process can only achieve a high yield of either high-APC heavy naphtha or low-BMCI tail oil (Alsobaai et al., 2007a; Looi et al., 2012).

Commercial hydrocracking catalysts use zeolite Y for the cracking reactions of C–C bond cleavages (Sato et al., 2001; Taufiqurrahmi et al., 2011). However, the highly acidic zeolite Y causes the over-cracking of intermediate products, leading to high yield of gas products and low yield of heavy naphtha (Cui et al., 2012a,b). The acidity of zeolite Y was reported to be reduced by chemical treatment by reducing the coke deposition on the hydrocracking catalyst (Gao et al., 2012; Zhou et al., 2013). Citric acid-treated zeolite Y (CY) exhibited reduced acidity of the strong Brönsted acid sites of zeolite Y due to the dissolution of a certain amount of aluminum from the zeolite Y structure (Wei et al., 2010). The use of zeolite beta as a hydrocracking catalyst support is known to promote isomerization reactions, resulting in the high yield of high-APC heavy naphtha (Horáček et al., 2012; Yao et al., 2012).

In this paper, various CY-zeolite beta (CY-Beta) composite catalysts were prepared by varying the mass ratios of zeolite CY and zeolite beta and used for vacuum gas oil (VGO) hydrocracking to produce high yields of high-APC heavy naphtha and low-BMCI tail oil.

## Experimental

### Materials

The VGO was obtained from the PetroChina Daqing Refinery. The properties of VGO are listed in Table 1. Zeolite Y and zeolite beta were obtained from the Sinopec Zhoucun Catalyst Factory and Nankai University Catalyst Company, respectively. Citric acid, nickel nitrate, ammonium meta-tungstate, ammonium molybdate, and cobalt nitrate were purchased from Sinopharm Chemical Reagent Company. All chemicals were used without further purification.

**Table 1:** Properties of vacuum gas oil (VGO).

**Properties**	
Density @ 15°C /g·mL^−1^	0.9138
Sulfur /μg·g^−1^	25.3
Nitrogen /μg·g^−1^	335
wt% distilled /°C	
0.5%	206
10%	338
20%	374
30%	397
40%	414
50%	428
60%	444
70%	460
80%	477
90%	496
95%	517

### Synthesis of Citric Acid-Treated Zeolite Y (CY)

The procedure used for the synthesis of CY has been described elsewhere (Wei et al., 2010). Zeolite Y was dispersed into 0.05 mol/L citric acid solution in a ratio of 1:10 (g/mL). The mixture was kept at 35°C for 1 h and then filtered through 25-μm filter paper. The CY on the filter paper was rinsed with deionized water and then dried in an oven at 120°C for 3 h.

CY-Beta was prepared by mixing CY and zeolite beta in stoichiometric ratios and then stocked for further use; the samples were named as CY-Beta(n), where n is the content of zeolite beta in the composite.

### Preparation of Hydrocracking Catalyst

Amorphous silica alumina (ASA) was prepared by mixing 30 mL of ammonia and 73 g of aluminum nitrate solution in a 500-mL reactor which at 60°C under continuous stirring followed by the addition of 25 g of sodium silicate. After 30 min, a white precipitate was obtained, and the ASA matrix was yielded after drying at 120°C for 4 h and calcinating at 500°C for 4 h.

The catalyst supports were prepared by mixing ASA and CY-Beta. The mixtures were extruded to form cylindrical extrudes with diameters of 1.5 mm. The Ni-W catalysts were prepared by the incipient wetness method, the supports were co-impregnated with 6 wt.% nickel oxide using nickel nitrate and 24 wt.% tungsten trioxide using ammonium meta-tungstate. The wet catalysts were dried at 120°C for 24 h and then calcined at 500°C for 4 h. The catalysts were designated as NiW/mCY-Beta(n)/ASA, where m is the content of CY-Beta in the support, and n is the content of zeolite beta in CY-Beta.

### Catalyst Characterization

Powder X-ray diffraction (XRD) was performed on the catalysts using a SIMADU XRD 6000 diffractometer with Cu Kα radiation operated at 40 kV and 40 mA.

The surface areas and pore size distributions of the catalysts were determined by nitrogen adsorption-desorption isotherms using a Micromeritics ASAP2450 instrument. Prior to adsorption testing, 30–40 mg of catalyst samples were degassed at 200°C under vacuum (1.33 ×10^−3^ Pa) for 15 h. The adsorption and desorption tests were carried out using liquid nitrogen at 77 K. The specific surface area of the catalyst sample was calculated using the Brunauer-Emmett-Teller (BET) method. The total volume of micropores and mesopores was calculated from the amount of nitrogen adsorbed at 0.98 P/P_o_. The micropore volume and external surface area were determined from the t-plot analysis. The pore size distribution of the catalyst sample was calculated with the N_2_ desorption isotherm using the Barrett-Joyner-Halenda (BJH) method.

The IR spectra of the catalyst samples were obtained using a Magna-IR 560 ESP infrared spectrophotometer with 1 cm^−1^ resolution. The catalyst sample was diluted in KBr (spectroscopy grade).

Scanning electron microscopy (SEM) measurements were performed on a Quanta 200 (FEI Co., Netherlands) apparatus combined with energy-dispersive X-ray spectroscopy (EDS) with an operation voltage of 20 kV. Prior to measurement, the samples were coated with a thin layer of gold.

The acid sites of the catalyst samples were determined by pyridine FT-IR (Py-IR) using a Magna-IR 560 ESP spectrophotometer with a resolution of 1 cm^−1^. The catalyst sample was ground and pressed into a wafer with a diameter of 12 mm and a thickness of 2–3 mm. The wafer was degassed in the IR cell at 350°C under vacuum (1.33 ×10^−3^ Pa) for 2 h. After the wafer was cooled to room temperature, the IR spectrum was recorded in the range of 1,700–1,400 cm^−1^. Subsequently, the wafer sample was subjected to the adsorption of pyridine vapor at room temperature for 20 min. The Py-IR spectra were recorded after the pyridine was evacuated from the IR cell at 200 and 350°C.

The sorption of ammonia (NH_3_) and temperature-programmed desorption (NH_3_-TPD) of the catalyst were measured using a Quantachrom Chembet 3,000 chemical adsorption instrument. The catalyst adsorbed NH_3_ at 120°C for 30 min after pretreatment at 500°C under 30 mL/min argon (Ar) purging for 1 h. The NH_3_ adsorbed physically at 120°C was removed by argon purging for 1 h. The NH_3_-TPD of the catalyst was carried out by the temperature-programmed heating of the catalyst from 120 to 750°C at 10°C/min. The NH_3_ desorption rate was determined from the nitrogen concentration in the effluent gas measured using a thermal conductivity detector (TCD).

### VGO Hydrocracking

VGO catalytic hydrocracking experiments were carried out in a 50-mL continuous fixed-bed reactor system. The tubular reactor was loaded with 10 mL catalyst having 0.59–0.84 mm particle diameters. The catalyst was pre-sulfided with a cyclohexane solution (2 wt% CS_2_) at 320°C for 4 h. With the exception of temperature, which was varied from 350 to 380°C, the hydrocracking reaction conditions were kept constant (4.0 MPa, 2.0 h^−1^ liquid hourly space velocity (LHSV), and 1,000 (V/V) hydrogen to oil ratio).

After 5 h of continuous hydrocracking, the catalyst was deactivated and reached an equilibrium state. The gas products were collected and analyzed using an Agilent 6890 gas chromatograph. The boiling point distribution of the liquid product was obtained by simulated distillation (SIMDIS ASTMD-2887). The total liquid product was fractionated into light naphtha (IBP-65°C), heavy naphtha (65–177°C), diesel (177–320°C), and tail oil (320°C^+^). The conversion and petrochemical feed yield are defined as follows:

(1) $$\begin{array}{rcl} {\;B.P.}_{177}\;conversion,\;\% & = & \left(1-\frac{{B.P.}_{177,product}}{{B.P.}_{177,feed}}\right)\times 100 \end{array}$$

(2) $$\begin{array}{rcl} Y_{j} & = & \frac{X\times S_{j}}{\sum X\times S_{j}} \end{array}$$

(3) $$\begin{array}{l} Petrochemical\;feed\;yield=light\;naphtha\;yield\\ \;+heavy\;naphtha\;yield\\ \;+\;tail\;oil\;yield \end{array}$$

Where Yj is the yield to j product, X is the conversion and Sj is the selectivity to j product.

The BMCI value was calculated using Equation 4 (Nyathi et al., 2012) to determine the quality of tail oil for steam-cracking feedstock. Poly-aromatic hydrocarbons have the highest BMCI (higher than 200), whereas paraffinic hydrocarbons have the lowest BMCI (<20):

(4) $$\begin{array}{r} BMCI=\frac{48460}{T}+473.7\times d-456.8 \end{array}$$

Where T is the volume average boiling point temperature (in K), and d is the density at 15.6°C (in g/mL).

The APC of naphtha was calculated using Equation 5 (ASTM D5580-02) (Han et al., 2014) to determine the quality of naphtha as a catalytic reforming feedstock:

(5) $$\begin{array}{l} APC\;=\;Benzene\;potential\;content\;+toluene\;potential\;content\\ \;+C8\;aromatic\;potential\;content \end{array}$$

In Equation 5:

(6) $$\begin{array}{l} \;Benzene\;potential\;content\\ =C6\;cycloalkanes\;content\times \frac{78}{84}+Benzene\;content \end{array}$$

(7) $$\begin{array}{l} \;Toluene\;potential\;content\\ =C7\;cycloalkanes\;content\times \frac{92}{98}+Toluene\;content\; \end{array}$$

(8) $$\begin{array}{l} \;C8\;aromatics\;potential\;content\\ =C8\;cycloalkanes\;content\times \frac{106}{112}+C8\;aromatics\;content \end{array}$$

Where all the contents listed above are in the units of mass ratio (m %).

The cetane index (CI) of diesel was calculated using Equation 9 (Aleme and Barbeira, 2012) to determine the quality of diesel as a transportation fuel:

(9) $$\begin{array}{l} CI=454.74-1641.416\times d+774.74\times d^{2}-1.554\times T\\ \;+97.8\times {\left(logT\right)}^{2} \end{array}$$

Where *d* is the density at 15.6°C (in g/mL), and *T* is the volume average boiling point temperature (in °C).

## Results and Discussion

### Properties of CY-Beta

**Figure 1:**
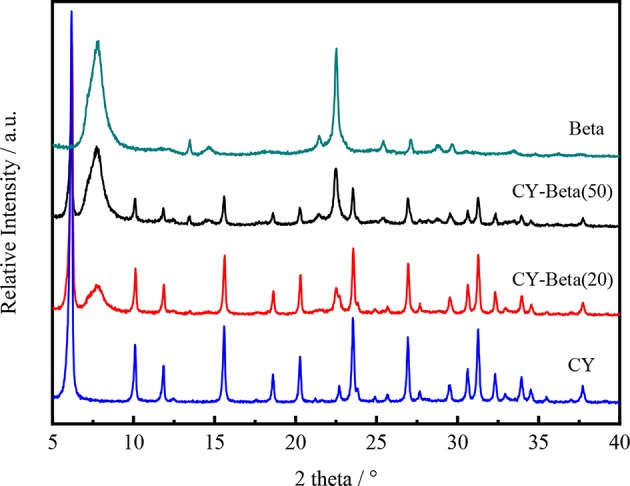
XRD patterns of zeolites CY, Beta, CY-Beta(20), and CY-Beta(50).

Figure 1 shows the XRD patterns of CY, zeolite beta and CY-Beta(n) with zeolite beta contents of 20 and 50. The XRD pattern of the CY-Beta composites exhibited the characteristic peaks of CY and zeolite beta, indicating the co-existence of these phases in the CY-Beta(n) composites. The peak intensities of CY in the CY-Beta composites varied as a function of the zeolite beta content in CY-Beta. The intensities of the zeolite beta peaks at 7.5° and 22.48° increased as the zeolite beta content increased.

**Figure 2:**
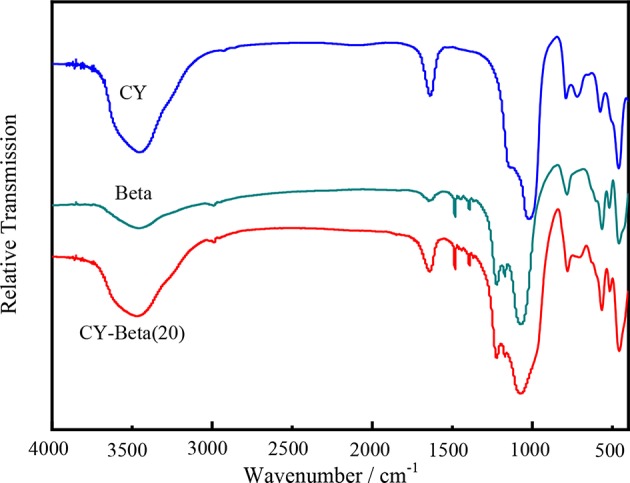
FT-IR spectra of zeolite CY, Beta, and CY-Beta(20).

Figure 2 shows the FT-IR spectra of CY, zeolite beta, and CY-Beta(20). The spectral band of zeolite CY at 460 cm^−1^ was assigned to the skeleton insensitive internal of MO_4_ tetrahedral bending peak of zeolite Y, where M is either Si or Al (Setiabudi et al., 2013; Tan et al., 2013). The band at 570 cm^−1^ was assigned to the double ring external linkage of zeolite Y (D'Ippolito et al., 2013). The bands at 685–775 cm^−1^ and 1,010–1,080 cm^−1^ were assigned to symmetry stretching and asymmetrical stretching of the inner tetrahedra of zeolite Y, respectively (Azzolina-Jury et al., 2013). The IR absorption bands in the high-energy region from 1,500 to 4,000 cm^−1^ are typical of symmetrical and asymmetrical C-H and Si-O stretching vibrations, whereas the low-energy bands were due to bond deformations. The stretching band at 3,450 cm^−1^ corresponds to the structural -OH groups, while that at 1,650 cm^−1^ was assigned to the scissor vibration arising from proton vibration in water (Newsam et al., 1988). The IR spectra also indicated that the zeolite samples had highly crystalline structures.

The absorption bands at 525, 573, and 623 cm^−1^ confirmed the presence of the zeolite beta structure (Li et al., 2009; Moller et al., 2011). The bands at 950–1,250 cm^−1^ were assigned to cross-out bonds and water hydroxyl bonds (Ausavasukhi et al., 2012; Horáček et al., 2012). The spectrum of zeolite beta showed a distinct band at 900–950 cm^−1^, indicating the terminal Si-O groups at the external surfaces of the crystallites (González et al., 2011; Laredo et al., 2012). These groups appeared in a high concentration of small crystallites (Li et al., 2009). In general, the FT-IR spectra of the synthesized CY and zeolite beta had similar skeletal vibration bands that are typical of zeolite absorption peaks.

For the CY-Beta composites, some of the bands of CY and zeolite beta were not apparent due to band overlap. The band locations of CY-Beta were the same as those of CY and zeolite beta, indicating that CY-Beta was a mixture of CY and zeolite beta.

Table 2 shows the BET N_2_ adsorption-desorption results of CY, beta zeolite, and CY-Beta(20). The specific surface area (*S*_BET_) of CY-Beta(20) was lower than that of zeolite Y and higher than that of zeolite beta. The micropore volume (*V*_mp_) of CY-Beta(20) was 0.15 mL/g, between the *V*_mp_ values of CY and zeolite beta. However, the mesopore volume (*V*_mep_) and total pore volume (*V*_t_) of CY-Beta(20) were higher than those of CY and zeolite beta. This shows that the mixing CY and zeolite beta did not destroy the zeolite structures, while more inter-granular pores were created.

**Table 2:** Typical properties of zeolites CY, Beta, and CY-Beta(20).

**Item**		**CY**	**Beta**	**CY-Beta(20)**	CY-Beta(20)^*^
Specific surface area /m^2^·g^−1^	S_BET_	467	304	385	434
	S_mep_	13	80	48	26
	S_mp_	454	224	330	408
Pore volume /cm^3^·g^−1^	V_t_	0.26	0.23	0.31	0.25
	V_mep_	0.03	0.11	0.16	0.05
	V_mp_	0.23	0.12	0.15	0.21
Average pore diameter/nm		0.37	0.36	0.37	0.37
Total acid	B	3.30	1.15	2.35	2.87
	L	3.41	5.52	3.86	3.83
	B/L	0.97	0.21	0.61	0.75
Strong acid	B	2.71	1.03	2.05	2.37
	L	2.90	5.26	3.34	3.37
	B/L	0.93	0.20	0.61	0.70

* *Calculated based on linear combination of zeolites Beta content is 20%*.

**Figure 3:**
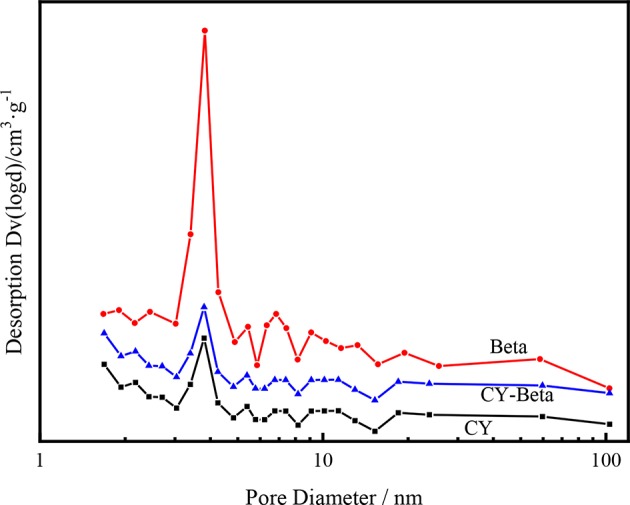
Pore diameter distributions of zeolites CY, Beta, and CY-Beta.

Figure 3 shows the BJH pore size distributions of CY, zeolite beta, and CY-Beta(20). The pore size distributions of these samples were centered at 3.7 nm, demonstrating that the CY-Beta composite was a micropore zeolite.

**Figure 4:**
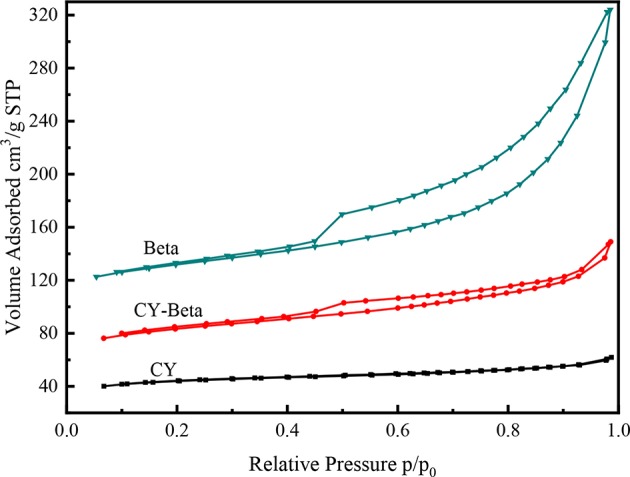
N_2_ adsorption-desorption isothermal curves of zeolites CY, Beta, and CY-Beta.

Figure 4 shows the N_2_ adsorption-desorption isotherms of CY, zeolite beta, and CY-Beta(20). CY exhibited an irreversible Type I adsorption isotherm typical of a microporous material, whereas zeolite beta exhibited a Type IV adsorption isotherm typical of a mesoporous material. The N_2_ adsorption for all zeolite samples started at a low relative pressure (P/P_o_ < 0.5) due to monolayer coverage of the micropore surface of the zeolite phase. The hysteresis loops of the N_2_ adsorption isotherms of zeolite beta and CY-Beta(20) were in the relative pressure range of 0.5–1.0, indicating the presence of textural mesopores, especially for CY-Beta(20).

The acidity of the catalyst plays an important role in the catalytic activity. Py-IR spectroscopy is often used to quantify the Brönsted and Lewis acid sites of the catalysts (Bonelli et al., 2007; Muddada et al., 2011). To identify the nature of the acid sites (Lewis and Brönsted acid sites) responsible for the cracking activity of the zeolites, samples with adsorbed pyridine were examined using FT-IR in the range of 1,400 to 1,700 cm^−1^ (Taufiqurrahmi et al., 2011).

**Figure 5:**
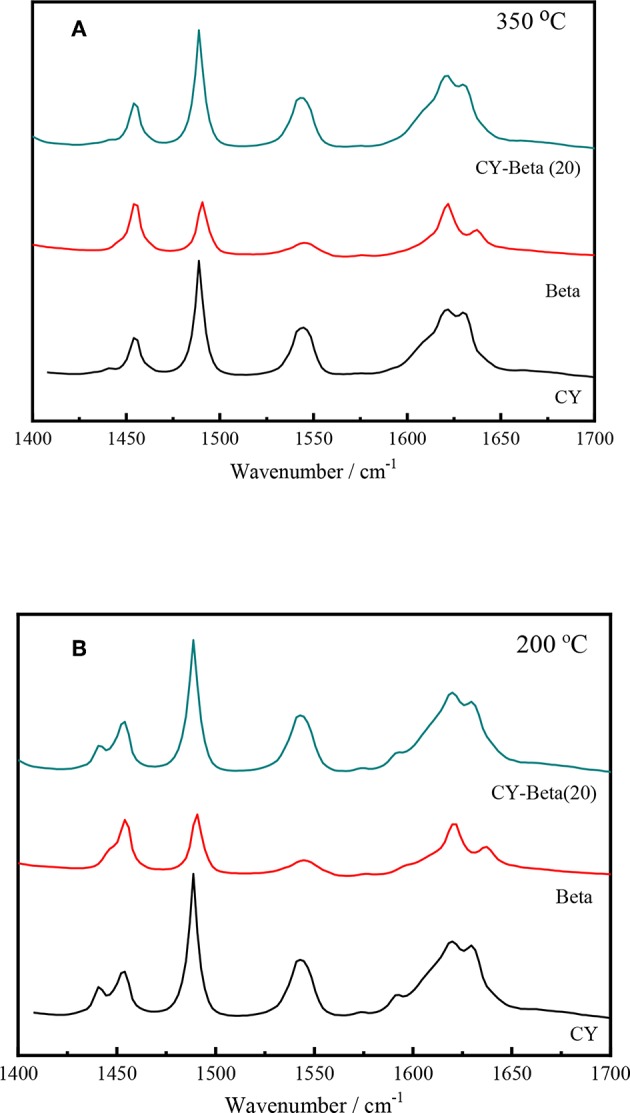
Py-IR spectra of zeolites CY, Beta, and CY-Beta(20) supported catalysts. **(A)** Degassed at 350°C, **(B)** Degassed at 200°C.

Figures 5A,B show the FT-IR spectra of CY, zeolite beta, and CY-Beta(20) after pyridine adsorption for samples. The IR system was degassed at 200 and 350°C, respectively, prior to pyridine adsorption. The 200 and 350°C spectra correspond to total acidity and strong acidity, respectively. The absorption bands at 1,490 cm^−1^ were attributed to pyridine adsorption on Lewis and Brönsted acid sites. The 1,445 and 1,545 cm^−1^ bands were assigned to the pyridine adsorption on Lewis and Brönsted acid sites, respectively. All samples also exhibited another Brönsted acid band at 1,638 cm^−1^. The calculated densities of Brönsted and Lewis acid sites on the surfaces of these three catalysts are summarized in Table 2. The density of acid sites on the CY-Beta(20) catalyst was a linear combination of the CY and zeolite beta. However, the total, medium, or strong acid sites in CY-Beta(20) were not linearly correlated with the calculated value. This is due to the destruction of acid sites exposed to the surface of the zeolite as a result of mechanical agitation during catalyst preparation.

**Figure 6:**
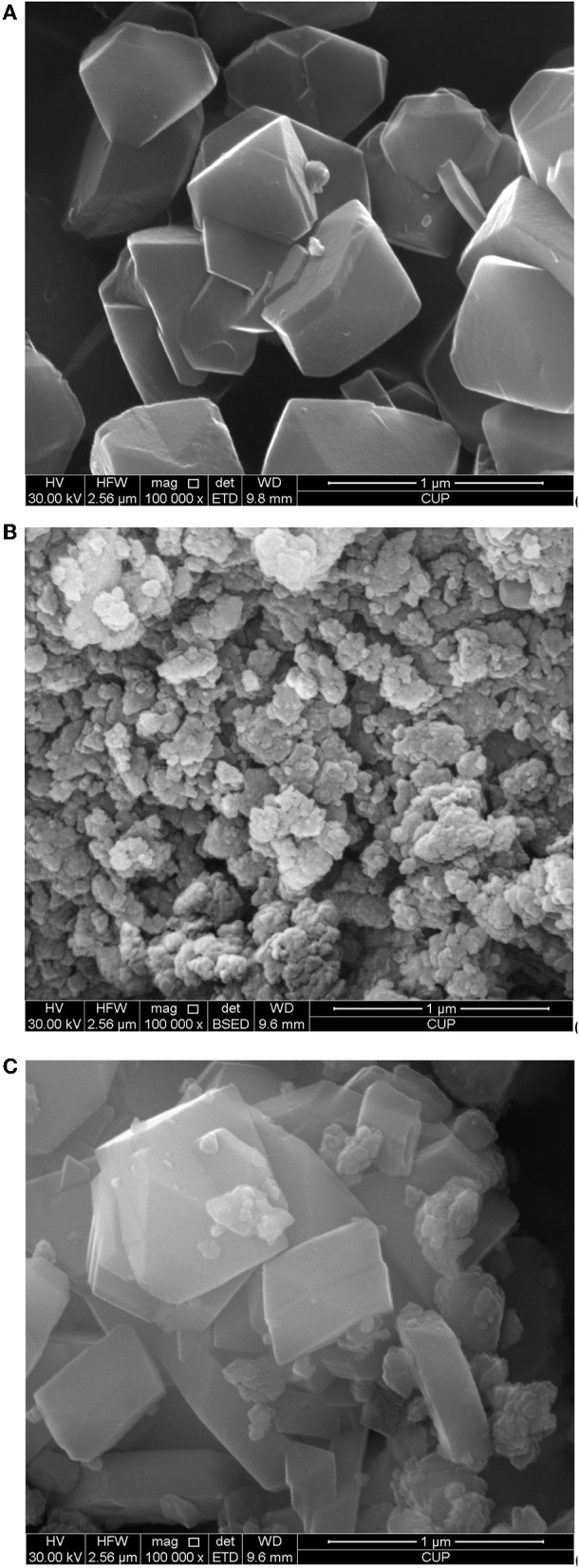
SEM micrographs of **(A)** zeolite CY, **(B)** zeolite Beta, and **(C)** zeolite CY-Beta(20).

Figures 6A–C show the crystallite sizes and morphologies of CY, zeolite beta, and CY-Beta(20) determined by SEM, respectively. CY consisted of relatively uniform crystals with sizes of 300–500 nm. Zeolite beta was in the form of agglomerates consisting of 100-nm particles with indistinct crystal structures. In CY-Beta(20), the zeolite beta particles attached on the outer surface of the CY crystals (Figure 6C). Figure 6C also shows that an intergranular structure was created by mixing CY and zeolite beta. It is suggested that the zeolite beta on the outer surface of CY favors the isomerization of intermediate cracking products diffused from CY (Newsam et al., 1988; Taufiqurrahmi et al., 2011; Alzaid and Smith, 2013).

### Properties of CY-Beta Supported Catalysts

**Figure 7:**
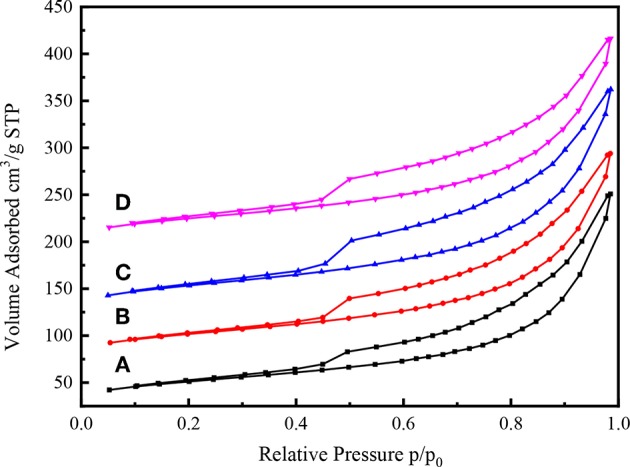
N_2_ adsorption-desorption isothermal profiles of **(A)** Ni-W/CY-Beta(50)/ASA, **(B)** Ni-W/CY-Beta(33)/ASA, **(C)** Ni-W/CY-Beta(20)/ASA, and **(D)** Ni-W/CY-Beta(15)/ASA.

The N_2_ adsorption-desorption isotherms of various Ni-W/CY-Beta(n)/ASA catalysts with zeolite beta contents varying from 15 to 50 are shown in Figure 7. All the catalysts exhibited Type IV isotherms typical of mesoporous materials with H-IV hysteresis loops, indicating the presence of slit-shaped pores (Mediavilla et al., 2013).

Similar to the N_2_ adsorption-desorption isotherms of CY-Beta(20) (Figure 4), the N_2_ adsorption of Ni-W/CY-Beta(20)/ASA catalysts started at a relative low pressure (P/P_o_ < 0.5) due to the monolayer coverage of the micropore surface of the zeolite phase (Habib et al., 2008). The hysteresis loops of the isotherms in the relative pressure range of 0.5–1.0 suggested the presence of textural mesopores. However, the sharp inflection regimes of the isotherms at 0.9 P/P_o_ were related to the diameter and uniformity of the mesopore of CY-Beta(20)/ASA support materials, indicating the presence of non-uniform mesopores in the support materials.

The specific surface areas, pore diameters, and volumes of various Ni-W/CY-Beta(n)/ASA catalysts are listed in Table 3. The ranges of these physical properties (176–189 m^2^·g^−1^ surface area, 8.4–8.8 nm pore diameter, and 0.38–0.41 cm^3^·g^−1^ pore volume) were favorable for the diffusion of reactants into the interior of the catalysts and enhanced the contact of reactants at the acid sites, avoiding excessive deep cracking of the reaction products. Since a high amount of ASA (80 wt% of catalyst mass) was used in preparing the Ni-W/CY-Beta(n)/ASA catalysts, most of the catalyst surfaces and pores were covered by ASA, and the CY/Beta mass ratio of the CY-Beta composite had little impact on the textual structure of resultant catalyst.

**Table 3:** Pore structure characterization of catalyst with various amounts of zeolite Beta.

**Item**	**Zeolite Beta concentration /%**	**Specific surface area /m^2^·g^−1^**	**Pore volume /cm^3^·g^−1^**	**Average pore diameter /nm**
Ni-W/CY-Beta(50)/ASA	50	176	0.39	8.8
Ni-W/CY-Beta (33)/ASA	33	178	0.38	8.5
Ni-W/CY-Beta (20)/ASA	20	189	0.41	8.6
Ni-W/CY-Beta (15)/ASA	14	178	0.38	8.4

The acid sites of the catalysts were quantified by NH_3_-TPD. Ammonia is a polarized molecule with a lone pair of electrons, which can be bonded to many type of materials (Huang et al., 2010). The adsorption of NH_3_ onto zeolites may result in the formation of a variety of chemically distinct species depending on the types of acid sites present within their porous structures (González et al., 2011).

**Figure 8:**
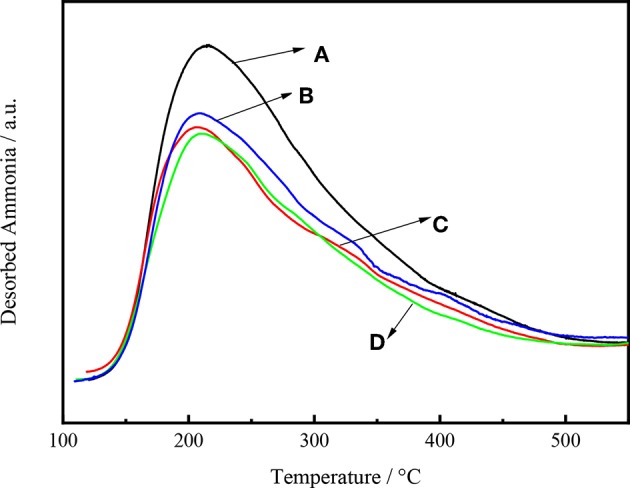
NH_3_-TPD profiles of catalysts **(A)** Ni-W/CY-Beta(50)/ASA, **(B)** Ni-W/CY-Beta(33)/ASA, **(C)** Ni-W/CY-Beta(20)/ASA, and **(D)** Ni-W/CY-Beta(15)/ASA.

Figure 8 shows the NH_3_ desorption as a function of temperature for Ni-W/CY-Beta(n)/ASA catalysts with various zeolite beta contents. All the NH_3_ desorption curves exhibited distinct maximum NH_3_ desorption peaks at 230°C, suggesting that the catalysts had weak acid sites (Wang et al., 2013). For Ni-W/CY-Beta(33)/ASA and Ni-W/CY-Beta(20)/ASA, weak peaks at 310°C were observed, suggesting that the catalysts also had certain moderate acid sites. In addition, the vertical shift in maximum NH_3_ desorption peak with decreasing zeolite beta content indicated that the number of acid sites on the catalyst increased with decreasing zeolite beta content.

**Figure 9:**
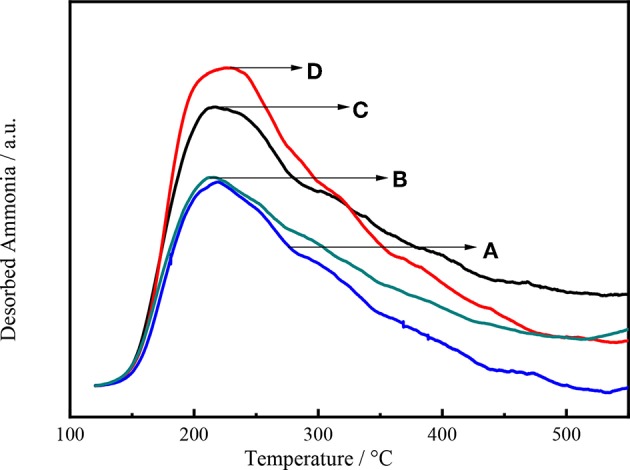
NH_3_-TPD profiles of **(A)** Ni-W/10CY-Beta(20)/ASA, **(B)** Ni-W/15CY-Beta(20)/ASA, **(C)** Ni-W/20CY-Beta(20)/ASA, and **(D)** Ni-W/25CY-Beta(20)/ASA catalysts.

Figure 9 shows the intensity of NH_3_ desorption as a function of temperature for Ni-W/mCY-Beta(20)/ASA catalysts with various CY-Beta(20) contents. The number of acid sites on the catalyst increased with decreasing CY-Beta(20) content. The catalyst with 20% CY-Beta(20) had the highest amount of acid sites in terms of both weak and strong acid sites. The peak locations were the same for all catalysts, indicating that the relative acid strengths were the same. This shows that the acid sites of composite zeolite were mainly weak and moderate acid sites. The weak acid sites are known to favor the production of low-BMCI tail oil, whereas numerous strong acid sites are beneficial for the production of highly-aromatic naphtha. Therefore, Ni-W/20CY-Beta(20)/ASA is likely a good hydrocracking catalyst.

### Hydrocracking Performance of CY-Beta Catalysts

Table 4 shows the results of continuous VGO hydrocracking pilot experiments with various Ni-W/20CY-Beta(n)/ASA catalysts. As the zeolite beta content increased, the hydrocracking conversions increased slightly, reaching a maximum at 20% zeolite beta, and then decreased. The light oil yield and petrochemical feed yield reached maxima of 66.0 and 83.7%, respectively, at a 20% zeolite beta content. Compared to the composite zeolite-supported catalysts, the CY and zeolite beta catalysts showed lower conversion. The high petrochemical feedstock yield was a result of the low hydrocracking conversion that induced the high yield of tail oil. This shows that the composite of CY and zeolite beta had some cooperative properties of the two zeolites and favored the production of high-APC naphtha and low-BMCI tail oil. The Ni-W/20CY-Beta(20)/ASA catalyst showed good hydrocracking activity and high light oil yield at 350°C, 8.0 MPa, 2.0 h^−1^ of LHSV, and 1,000 H_2_/oil (v/v).

**Table 4:** Hydrocracking results of Ni-W/mCY-Beta(n)/ASA catalysts.

**Item**	**Conversion /%**	**Light oil yield /%**	**Petrochemical feed yield /%**
Ni-W/20Beta/ASA	73.21	54.6	75.2
Ni-W/20CY-Beta(50)/ASA	76.76	61.4	81.0
Ni-W/20CY-Beta(33)/ASA	77.92	63.6	82.2
Ni-W/25CY-Beta(20)/ASA	77.93	63.8	82.8
Ni-W/20CY-Beta(20)/ASA	78.15	66.0	83.7
Ni-W/15CY-Beta(20)/ASA	77.70	63.6	82.1
Ni-W/10CY-Beta(20)/ASA	74.54	59.8	81.9
Ni-W/20CY-Beta(15)/ASA	77.61	64.6	82.0
Ni-W/20CY/ASA	74.34	60.4	85.9

*Reaction conditions: 350^o^C, 8.0 MPa, 2.0 h^−1^, 1,000 (v/v)*.

Table 5 shows the hydrocracking performance of catalysts supported by the CY-Beta(20) composite [Ni-W/10CY-Beta(20)/ASA, Ni-W/15CY-Beta(20)/ASA, Ni-W/20CY-Beta(20)/ASA, and Ni-W/25CY-Beta(20)/ASA]. By increasing the composite zeolite content, the hydrocracking conversion, light oil yield, and petrochemical yield increased gradually, reaching a maximum at 20% zeolite content, and then decreased. The maximum hydrocracking conversion, light oil yield, and petrochemical yield were 78.15, 65.0, and 83.7%, respectively. The properties of the Ni-W/20CY-Beta(20)/ASA catalyst-derived hydrocracking products show that the BMCI of the tail oil was 4.7, and the APC of heavy naphtha was 42%. This showed that the tail oil was a high quality petrochemical feedstock for steam cracking to produce ethane, and heavy naphtha was a high quality feedstock for the reforming processing of aromatics.

**Table 5:** Reaction results of catalysts with different amounts of zeolite.

**Catalyst**	**Conversion, %**	**Light oil yield, %**	**Petrochemical feed yield, %**
Ni-W/10CY-Beta(20)/ASA	74.54	59.8	81.9
Ni-W/15CY-Beta(20)/ASA	77.70	63.6	82.1
Ni-W/20CY-Beta(20)/ASA	78.15	65.0	83.7
Ni-W/25CY-Beta(20)/ASA	77.93	63.8	82.8

*Reaction conditions: 350^o^C, 8.0 MPa, 2.0 h^−1^, 1,000 (v/v)*.

The Ni-W/20CY-Beta(20)/ASA catalyst was subjected to continuous pilot plant testing to determine its equilibrium catalytic activity at 350°C, 7.0 MPa, 2.0 h^−1^ LHSV, and 800 H_2_/oil (v/v). Table 6 shows that the catalyst activity remained stable during a 1,500 h pilot run, with relatively constant conversion, liquid yield, liquid product distribution, and petrochemical yield. The yield of heavy naphtha was 41.2 with an APC of 41.0%, and the yield of tail oil was 14.9 with a BMCI of 4.4. The yield of diesel was about 25% with a CI of 59.2; the freezing point was lower than −45°C, and the cold filter plugging point was −35°C. The high APC of heavy naphtha suggested that the catalysts had aromatization and cyclization functions. The low freezing point and filter plugging point of diesel showed the isomerization function of the catalyst. The low BMCI of the tail oil showed the high catalytic hydrogenation activity for transforming un-cracked tail oil to saturated hydrocarbon.

**Table 6:** Aging test of Ni-W/20CY-Beta(20)/ASA.

		**Ni-W/20CY-Beta(20)/ASA**		
Sampling time, hours on stream		500	1,000	1,500
>177°C fraction conversion /%		60.2	59.8	59.8
${\text{C}}_{5}^{+}$ liquid yield /%		95.7	95.6	95.7
**REACTION CONDITIONS**				
Reaction temperature /°C		350	350	350
Operating pressure /MPa		7.0	7.0	7.0
H_2_/oil (v/v)		800	800	800
LHSV /h^−1^		2.0	2.0	2.0
**PRODUCT YIELDS AND PROPERTIES**				
IBP-65°C light naphtha /%		18.8	18.8	18.6
65–177°C heavy naphtha /%		41.4	41.0	41.2
	Aromatics potential content (APC) /%	40.4	41.8	41.4
177–320°C diesel /%		25.0	25.3	25.3
	Cetane index	59.1	59.2	59.2
	Frozen point/°C	< –45	< –45	< –45
	Filter plugging point / °C	−35	−35	−35
>320°C tail oil /%		14.8	14.9	14.9
	BMCI index	4.4	4.3	4.4
Petrochemical feedstocks /%		75.0	74.7	74.7

## Conclusions

This paper studied the properties of CY-Beta zeolite composites and the hydrocracking performances of the corresponding catalysts. The mechanical mixing of CY and zeolite beta does not destroy the textual properties of the zeolites. However, the acidity of the composite zeolite does not agree with the linearly calculated value of the two zeolites because some of the acid sites are covered or reacted during the mixing process. Furthermore, the presence of more weak acid sites favors the high yield of tail oil with a low BMCI value.

The mixing of CY and zeolite beta increases the conversion and light oil yield of the catalyst compared to CY-based and zeolite beta-based catalysts. The conversion, light oil yield, and petrochemical yield of the Ni-W/20CY-Beta(20)/ASA catalyst are 78.15, 65.0, and 83.7%, respectively. The BMCI value of the tail oil is 4.7, and the APC of heavy naphtha is 42%.

The 1,500 h pilot plant test of Ni-W/20CY-Beta(20)/ASA at 350°C, 7.0 MPa, 2.0 h^−1^ LHSV, and 800 H_2_/oil (v/v) shows that activity remains stable during the 1,500 h evaluation. The yield of heavy naphtha is 41.2, with an APC of about 41.0, illustrating that the catalyst had aromatization and cyclization abilities. The yield of diesel is about 25%, with a CI of 59.2, a freezing point lower than −45°C, and a cold filter plugging point of −35°C, demonstrating the isomerization performance of the catalyst. The yield of tail oil is 14.9% with a BMCI of 4.4, indicating the high hydrogenation performance of the catalyst to transform un-cracked tail oil to saturated hydrocarbon in order to reduce the BMCI value.

## Data Availability Statement

All datasets generated for this study are included in the article/supplementary material.

## Author Contributions

QW supervised the experiments and draft the manuscript. SW carried out CY zeolite synthesis. YW carried out composite zeolite synthesis. ZZ carried out hydrocracking catalysts preparation. TZ carried out hydrocracking test. YZ supported the whole experiments. JZ revised the manuscript. XL supplemented experiments. PZ carried out charaterizations.

### Conflict of Interest

SW, YW, ZZ, and TZ were employed by company China National Petroleum Corporation. The reviewer AD declared a shared affiliation, though no other collaboration, with the authors QW, JZ, XL, PZ. The remaining authors declare that the research was conducted in the absence of any commercial or financial relationships that could be construed as a potential conflict of interest.
